# Cochrane corner: artificial intelligence for keratoconus

**DOI:** 10.1038/s41433-024-03347-z

**Published:** 2024-09-19

**Authors:** Ethan Wen Wei Tiong, Su-Hsun Liu, Darren S. J. Ting

**Affiliations:** 1 School of Medicine, University of Manchester, Manchester, UK; 2 Department of Ophthalmology and Department of Epidemiology, University of Colorado Anschutz Medical Campus, Aurora, CO USA; 3 Academic Unit of Ophthalmology, Institute of Inflammation and Ageing, College of Medical and Dental Sciences, University of Birmingham, Birmingham, UK; 4 Birmingham and Midland Eye Centre, Sandwell and West Birmingham NHS Trust, Birmingham, UK; 5 Academic Ophthalmology, School of Medicine, University of Nottingham, Nottingham, UK; 6 Singapore Eye Research Institute, Singapore, Singapore

**Keywords:** Corneal diseases, Diagnosis

## Introduction

Keratoconus is a bilateral, progressive and asymmetrical disease which causes cone-like corneal steepening and irregular astigmatism [1, 2]. It is the most common corneal ectasia worldwide, and a common indication for corneal transplantation [3]. Important risk factors include eye rubbing, atopy, family history of keratoconus, amongst other host, genetic and environmental factors [4]. Keratoconus typically presents in the second to third decades of life and affects all genders and ethnicities [2], with higher prevalence rates observed in Middle Eastern and Asian populations [2].

Corneal topography/tomography is currently the gold standard for screening, diagnosing and monitoring keratoconus [1]. This can be aided by several advanced analytical techniques such as Belin Ambrosio Enhanced Ectasia Display (for keratoconus detection) and Belin ABCD progression display (for monitoring keratoconus progression). Over the past decade, there has been a surge in interest in artificial intelligence (AI) for diagnosing various ophthalmic conditions, including keratoconus, but high-quality evidence is limited [5].

## Summary of cochrane review findings

A recently published Cochrane review by Vandevenne et al. [6] analysed 63 diagnostic test accuracy (DTA) studies from 1994 to 2022, evaluating the performance of AI algorithms (the index test) in diagnosing keratoconus. These studies encompassed 3843 participants (23,771 eyes) and 38,832 images, and used topography-, tomography-, or OCT-based imaging to diagnose manifest, subclinical, or mixed keratoconus. The reference standard was clinical diagnosis made by ≥1 clinicians with corneal imaging, though the details were lacking in many studies. AI algorithms exhibited an overall high accuracy in detecting manifest keratoconus, with a summary sensitivity of 98.6% (95% CI 97.6–99.1) and specificity of 98.3% (97.4–98.9). Accuracy for diagnosing subclinical keratoconus was reduced, with a summary sensitivity of 90.0% (84.5–93.8) and a specificity of 95.5% (91.9–97.5); few studies reported AI algorithms for mixed (both subclinical and manifest) keratoconus (sensitivity 96.2% [92.5–98.1], specificity 98.0% [92.6–99.5]). The certainty of evidence for AI algorithms in the detection of both keratoconus subtypes was low due to potential risk of bias. Subgroup analysis found no significant differences in accuracy across study designs, AI algorithms, imaging techniques, or data sources. The heterogeneity and inconsistency across studies further contributed to the uncertainty of the evidence. The review authors suggested that future studies should maximise sensitivity over specificity to improve the detection of subclinical keratoconus and to prevent iatrogenic ectasia post-refractive surgery [6].

This review highlighted inadequate reporting on the study design, particularly regarding the reference standard, participants’ characteristics, and intra-study model comparisons when multiple candidate algorithms were developed. Notably, no study was judged to have low risk of bias or low concerns of applicability across all evaluated domains of the Revised Tool for the Quality Assessment of Diagnostic Accuracy Studies (QUADAS-2) [7]. Patient selection was the major source of potential risk of bias due to the overreliance on the case-control (two-gate) designs in the included studies (58/63, 92% of studies) [8]. Furthermore, the study populations often mismatched authors’ predefined criteria. The unit of analysis was also inconsistent; some studies analysed multiple images per eye while others reported performance results at the individual eye or participant level. There was also a lack of external validation in the included primary studies; except for one [9]. In nearly half of the included studies (30/63, 47.6%), the method for setting the reference standard was unclear.

## Need for improvement in AI-DTA studies

This review revealed notable gaps in the reporting by both primary studies of AI in keratoconus and the review process itself. As AI-DTA studies proliferate, detailed and transparent descriptions of methodology and decision-making processes are crucial for critical appraisal. For primary studies, researchers should follow established AI-specific checklists such as the Checklist for Artificial Intelligence in Medical Imaging (CLAIM) [10] and the CONSORT-AI standards for clinical trials [11]. Although still in development, the STARD-AI [12] and QUADAS-AI DTA [13] guidelines as well as the Cochrane DTA-AI extension protocol (currently in progress) will prove valuable in this regard. Encouraging external validation of AI diagnostic algorithms or “random” or “consecutive” sampling from the target population may produce more representative and clinically applicable results in real-world settings.

Effective evidence synthesis for systematic reviews remains challenging due to study heterogeneity and potential reporting biases among primary DTA studies. Given the complexity of DTA systematic reviews, adherence to the PRISMA-DTA guidelines will facilitate transparent reporting and minimise bias at the review level [14]. Clinicians should be cognisant of potential risks of biases arising from multiple domains within both primary studies and reviews, interpreting study findings cautiously.

## Expanding horizons of AI applications for keratoconus

Few AI algorithms for keratoconus detection have been externally validated and implemented in clinical practice. Notable exceptions include the Pentacam Random Forest Index [15] on the Oculus Pentacam® (OCULUS GmbH, Wetzlar, Germany) which uses tomographic data, and the Corneal Biomechanical Index [16] on the Corvis ST® (OCULUS GmbH, Wetzlar, Germany) which uses corneal biomechanical data instead. The Tomographic-Biomechanical Index (TBI) integrates both tomographic and corneal biomechanical data from the Pentacam® and Corvis ST® [17], with its recently optimised version (TBIv2) significantly outperforming its predecessor in detecting subclinical ectasia (AUC 0.945, 84.4% sensitivity, 90.1% specificity) [18]. Further improvements may be possible with corneal epithelial thickness measurements or multimodal refractive imaging [18].

In addition to diagnosis, several AI models have been developed to predict keratoconus progression using various data modalities, including tomographic images [19] and tomographic parameters [20] from Pentacam® scans, as well as colour-coded maps from swept-source anterior segment OCT [21]. A non-imaging approach incorporated environmental and ocular surface risk factors as well as biomarkers [22]. Other innovations include a portable smartphone-based keratoconus detection method with a 3D-printed gadget [23] and wearable sensors for prevention of eye rubbing [24].

## Conclusions

From keratoconus detection to innovative approaches in predicting disease progression and monitoring risk factor, AI demonstrates immense potential to enhance keratoconus management [5]. However, the present review highlights that the current landscape of AI research in this field is marked by inadequate reporting and methodological limitations. Future studies should prioritise representative study populations, rigorous external validation, and adherence to AI-specific reporting guidelines to allow for high-quality evidence. Addressing these challenges will be vital in translating promising research findings into reliable and applicable clinical tools.
